# Disagreeing in context

**DOI:** 10.3389/fpsyg.2015.00257

**Published:** 2015-03-19

**Authors:** Teresa Marques

**Affiliations:** Law Department, Philosophy of Law Area, Universitat Pompeu FabraBarcelona, Spain

**Keywords:** contextualism in semantics, disagreement, conflicting attitudes, de nobis attitudes, dispositional evaluative properties

## Abstract

This paper argues for contextualism about predicates of personal taste and evaluative predicates in general, and offers a proposal of how apparently resilient disagreements are to be explained. The present proposal is complementary to others that have been made in the recent literature. Several authors, for instance (López de Sa, 2008; Sundell, 2011; Huvenes, 2012; Marques and García-Carpintero, 2014; Marques, 2014a), have recently defended semantic contextualism for those kinds of predicates from the accusation that it faces the problem of lost disagreement. These authors have proposed that a proper account of the resilient disagreement in the cases studied is to be achieved by an appeal to pragmatic processes, and to conflicting non-doxastic attitudes. It is argued here that the existing contextualist solutions are incomplete as they stand, and are subject to objections because of this. A supplementation of contextualism is offered, together with an explanation of why failed presuppositions of commonality (López de Sa), disputes over the appropriateness of a contextually salient standard (Sundell), and differences in non-doxastic attitudes (Sundell, Huvenes, Marques, and García-Carpintero) give rise to conflicts. This paper claims that conflicts of attitudes are the reason why people still have impressions of disagreement in spite of failed commonality presuppositions, that those conflicts drive metalinguistic disputes over the selection of appropriate standards, and hence conflicting non-doxastic attitudes demand an explanation that is independent of those context dependent pragmatic processes. The paper further argues that the missing explanation is 2-fold: first, disagreement prevails where the properties expressed by taste and value predicates are response-dependent properties, and, secondly, it prevails where those response-dependent properties are involved in evolved systems of coordination that respond to evolutionarily recurrent situations.

## 1. Introduction

When people have disagreements about taste, or about aesthetic or moral values, what is their disagreement about? What explains the apparent fact that it is legitimate for people to hold on to their views about the issue under discussion? And what explains that the disagreements at stake are often resilient and persistent? Is there an account of this kind of disagreement that can capture the perspective dependence of a given domain while preserving the sense of resilient disagreement between those with different perspectives?

In the recent debate that has opposed contextualists to relativists about predicates of personal taste, aesthetics, and morality, contextualists have tried to resist objections raised by non-indexical contextualists and assessment-relativists by adopting two distinct strategies. The first strategy is to argue that none of the relativist positions now available fare better than contextualist ones^1^. The second strategy is to show how resilient disagreements are to be explained. On the one hand, contextualists have appealed to a combination of pragmatic mechanisms to account for these disagreements: presuppositions of commonality,^2^ and to further metalinguistic considerations about the choice of salient standards^3^. Contextualists have also added a more thorough explanation of the practical dimension of the disagreements at stake, for instance appealing to conflicts of non-doxastic attitudes^4^. Neither of these approaches—the pragmatic or the attitudinal—have been sufficiently developed so far. In this paper, I will indicate which aspects are still wanting. What is required is an account that frames both the pragmatic and the conative aspects within an explanation of inter-subjective or group coordination. The paper further argues that the missing account is 2-fold: first, disagreement prevails where the properties expressed by taste and value predicates are response-dependent properties, and, secondly, it prevails where those response-dependent properties are involved in evolved systems of coordination that respond to evolutionarily recurrent situations.

This paper is structured as follows. In Section 2, I present and indicate what is lacking in the otherwise promising contextualist proposals mentioned here. Thus, in Section 2.1, I show that appealing to presuppositions of commonality by itself is insufficient, because in other similar cases the awareness that a presupposition fails dispels the impression of disagreement. In Section 2.2, I consider Sundell's suggestion that the disputes take place at a metalinguistic level, and that in some of the relevant cases what is at stake is the choice of a salient standard. One problem with this proposal is that we need a better understanding of how disputes of this sort are to be adjudicated, and of what motivates speakers to pursue them. A further problem for both pragmatic explanations is that we have the impression that there are disagreements between subjects who are not part of the same conversational setting, or do not even interact in any form. Both presuppositions of commonality and metalinguistic disputes seem to require that some interaction exists. In Section 2.3, I raise a problem for solutions that rely on the incompatibility of (pro) attitudes. The most plausible explanation for the source of conflict—preclusion of joint satisfaction—seems not to yield the desired result. Nonetheless, I think these three proposals made on behalf of contextualism are basically correct.

Section 3 offers the beginning of a solution. In Section 3.1, I suggest that we should follow Lewis and Hume in treating practical agreements as solutions to coordination problems. Disagreements would arise when people's dispositions are obstacles to coordination. The suggestion is supported by research on group action and rationality. In Section 3.2, I offer a conjecture that can resolve the objections raised against contextualism. The main problems are, first, that we have impressions of disagreement even where subjects do not share a conversational setting, do not know of each other, and do not have common goals. Second, we have impressions of disagreement even when the apparent dispositions revealed in the disagreement can be satisfied. My conjecture is that the kind of coordination problems that the different types of dispute pose are at the root of our having, as humans, evolved to have the emotional responses we have, to make value judgments about matters of taste, aesthetics or morality, and, crucially, to hear conflicts in the expressions of different personal preferences. This section reviews some research that corroborates this conjecture.

In Section Section 4, I examine the consequences of the proposal offered here for the current debate between contextualists and relativists. First, disagreement prevails where the properties expressed by taste and value predicates are response-dependent properties, and, second, it prevails where those response-dependent properties are (i) *de nobis*;^5^ and (ii) involved in evolved systems of coordination that respond to evolutionarily recurrent situations.

## 2. Contextualist strategies

Why be a contextualist in the first place?

Contextualism (also called “indexical relativism” by Kölbel (2004), or “indexical contextualism”) is a semantic thesis. A contextualist about a given class *k* of expressions holds that an utterance *u* of a sentence *S* where a *k*-expression occurs as made at a given context *C* expresses a proposition *p* at *C* that is evaluated with respect to < *w* >_*C*_, the world of the context *C*. The class *k* is composed of expressions whose characters compositionally determine in context what content or proposition is expressed by an utterance of a sentence that contains a *k*-expression, *and* the content determined varies from context of use to context of use. The content or proposition expressed is then a function from possible circumstances of evaluation to extensions (in the case of sentences, to truth-values).

As Kölbel (2004) says, contextualists allow for different utterances of the same sentence to express different contents. An utterance expresses a given proposition as its content, and that content's truth will only be relative to possible worlds (as standard semantic theories require). Truth-relativists (moderate, i.e., non-indexical relativists, or radical, i.e., assessment-relativists), allow for the truth of the content expressed in context to depend on more than (just) a possible world.

Contextualism has both a linguistic and a metaphysical motivation. Speakers' judgments and *linguistic intuitions* are normally used in favor of contextualism in the various domains where contextualism has been defended, in particular judgments concerning:

- What is said;
- Whether what is said is true or false;
- On disagreement between people making different claims within any of these domains.

Yet, the available data on speaker's intuitions is not, at least not conclusively, decisive for contextualism, as relativist objections make clear. In the current debate about the meaning of predicates of personal taste and other evaluative predicates, several authors have raised objections against contextualist approaches, mainly on the basis that contextualism misses intuitions of disagreement that these writers show we have. The problem, as they argue, is that of lost disagreement.

Thus, Kölbel (2004) argues on this basis for what is usually called “moderate truth-relativism” (also known as “non-indexical contextualism,” by contrast with the more standard contextualist views). Egan (2010), Lasersohn (2005), and MacFarlane (2014) have argued for another version that can be called “assessment-relativism.” As this paper is not dedicated to discussing the limitations that either form of relativism may have in accounting for disagreement, I will not explain here the differences between these views. In any case, further arguments must be provided to settle this discussion, preferably arguments that assume some common ground with relativists. By framing the discussion within a broad dispositionalist metaphysical theory, I am sharing at least this common ground with relativists^6^.

There are good reasons for a *relational metaphysical* account of the properties expressed by predicates like “is funny,” “is disgusting,” “is tasty,” “is beautiful,” “is good,” etc. It seems highly implausible that claims about, for example, humor, taste, aesthetic value, and perhaps moral value, should be independent of how people react to funny, disgusting, tasty, or beautiful things.

An analogy with other dispositional properties can be helpful in understanding the motivation for a relational account of the relevant evaluative properties. Recently, Cohen (2009) argued that a metaphysical view of this kind about colors has a natural contextualist semantic implementation. Cohen draws attention to the fact that a single color stimulus can produce multiple psychophysically distinguishable perceptual effects in respect of color. He further adds that there is no well-motivated reason for thinking that just one of those variants is veridical. Thus, he concludes, predicates like “red” express relational properties, more specifically “response-dependent” ones such as *looking red to subjects of kind* S *under circumstances* K. By analogy with the color case, we can say that aesthetic and taste predicates—and perhaps moral predicates—express relational properties. A predicate like “is tasty,” or “is disgusting,” uttered in context *C*, expresses properties such as *tasty for the perceivers relevant in context* C *under the perceptual circumstances relevant in* C, or simply *tasty for the standard relevant in* C.

For the rest of the paper, I will assume that a dispositional account of the properties expressed by many evaluative predicates is correct, and that aesthetic, taste, humor, and moral predicates express relational properties. Saying this is not settling *who* the “subjects of kind *S*” are for each relational property. In some cases, one may expect universality (everyone) and in other cases expectations of universality might be unjustified.

It does not follow that any possible claims in matters of taste, or morality, exhibit such variability, and the extent to which there is any variability at all may vary between domains. Perhaps there is more variability in claims on matters of taste, and less in morality. Concrete sociological, historical or anthropological analysis would need to corroborate the actual degree to which such a variability exists.

The aim of this paper is to show that a contextualist can explain the resilient cases of disagreement, and, in so doing, take the wind out of the relativist's sails. The remainder of this section reviews three ways for a contextualist to secure disagreement: presuppositions of commonality, metalinguistic disputes, and conflicts of non-doxastic attitudes.

### 2.1. Presuppositions of commonality

López de Sa (2008) and López de Sa (2015) defends contextualism (indexical relativism) from criticism based on disagreement data by pointing out that the proper semantic implementation of the proposal should envisage the presuppositions of commonality that assertions expressing judgments of taste carry. According to him, the failure of these presuppositions accounts for the data. The main problem with López de Sa's proposal, as I see it, is that when presuppositions of the kind he envisages fail, we should not feel that any relevant disagreement remains. This is corroborated in the case of gradable adjectives like “rich” or “tall.” But a strong impression of disagreement is still felt even by semantically enlightened speakers, which cannot be explained by semantically blind folk invariantist intuitions.

Consider the following exchange between Clarissa and Jennifer, both excellent cooks with vast experience and good taste^7^.

1. (a) Clarissa: Cow's tongue is disgusting.
    (b) Jennifer: No, it's not disgusting; it's delicious.

People feel that Clarissa and Jennifer straightforwardly disagree. On contextualist semantics, however, if the relevant standard of taste is subject-relative, in their context the claims are equivalent to these:^8^

2. (a) Clarissa: Cow's tongue is disgusting [given Clarissa's standards].
    (b) Jennifer: No, it's not disgusting, it's delicious [given Jennifer's standards].

There seems to be no impression of disagreement in (2). In fact, as Kölbel (2004) points out, now both speakers can rationally accept what the other has said while maintaining their respective assertions, unlike what seemed to be the case in (1).

These are cases of what Egan, (2010, p. 251) calls first-personally committed (auto-centric) uses, to be distinguished from sympathetic (exocentric) uses in which we ascribe tastes by adopting alien perspectives (“that fodder must be delicious”).

Yet, the contextualist acknowledges that there must be cases of pointless disputes, where subjects have contrasting sensibilities. Subjects are thereby either expressing different relational properties (or wrongly purporting to express an inexistent one shared by both of them). (1) is an example of such a case of a “faultless” dispute—one that does not involve any doxastic disagreement over a unique context-dependent content that Clarissa accepts and Jennifer rejects. But how will the contextualist explain the persisting intuitions of disagreement concerning such cases?

López de Sa's explanation (López de Sa, 2008 pp. 304–305) appeals to presuppositions of commonality. The relevant predicate “triggers the presupposition that the participants in the conversation are similar” with respect to the relevant standard. López de Sa assumes a Stalnakerian account of presuppositions (cf. Stalnaker, 2002). On this account, presuppositions are requirements on the “common ground” (the class of propositions that participants in the conversation take to be known by all, known to be known by all, etc) that may be triggered by specific expressions or constructions. Utterances carrying the presuppositions are not felicitous unless the common ground includes them, or, if it does not, they are “accommodated” by the conversational participants, i.e., included in the common ground as a result of the utterance.

Impressions of disagreement in (1) are then explained because “in any non-defective conversation… it would indeed be common ground” that the participants are relevantly alike. In such a conversation, one would be right and the other wrong. Of course, in (1) the presupposition fails, and as a result both claims are infelicitous. In other words, the impression of disagreement is to be explained by the fact that the following conditional is true about (1): had Clarissa and Jennifer been in a felicitous context in which the presupposition of a common standard was met, then they would have disagreed^9^.

But impressions of disagreement in analogous cases also disappear, as witnessed by the case of the vagueness-inducing relativity to “perspectives” or “ways of drawing the line” for gradable adjectives, as the example offered below illustrates^10^. However, such impressions remain among the fully reflective in the case of judgments of taste like the ones considered here. The comparison with gradable adjectives shows that the presuppositional account does not help.

The next example (originally from Richard, 2004) suggests the indexicality of gradable adjectives—adjectives that admit comparative and superlative degrees, intensifiers like “much” and “very,” and so on, and illustrates how an impression of disagreement should disappear once different standards of wealth are made explicit. Imagine that Mary wins a million dollar lottery. Didi is impressed; but for Naomi, a million dollars is not much. Taking New Yorkers to be the relevant fields of comparison, they judge:

3. (a) Didi: Mary is rich.
    (b) Naomi: Mary is not rich.

The information about differential standards of richness provided by context, which accounts for the intuition that different contents are being affirmed and denied in (3a) and (3b), can in some other cases be explicitly articulated in the uttered sentence:

1. (a) Didi, as before: Mary is rich (given Didi's standard).
    (b) Naomi, as before: Mary is not rich (given Naomi's standard).

This evidence can be handled by means of a contextualist proposal, following suggestions about the semantics of gradable adjectives in the literature. Assuming that the speaker's intentions play a crucial role in determining degree significance, Didi and Naomi either do not disagree, or participate in an infelicitous conversation where *presuppositions of commonality* fail. The problem for López de Sa's proposal is that the impression of disagreement also vanishes among semantically enlightened speakers in this case. However, his counterfactual still applies: Didi and Naomi would be disagreeing, if they were speaking in a felicitous context. Didi's possible reply to (3b) illustrates this.

4. (a) Didi: Mary is rich given what counts as rich for me; I see that you have a different perspective on these matters.

Therefore, what explains the impression of resilient disagreement between Clarissa and Jennifer in (1) cannot be that a counterfactual of that sort applies. A proposal along the lines of López de Sa's might be the beginning of an explanation of such a perception of disagreement. However, as (4) and (5) show, the presence of presuppositions of commonality is not enough to explain the perception of disagreement that remains even for the semantically enlightened subjects who adopt a contextualist semantics for value predicates^11^.

### 2.2. Metalinguistic disputes

Sundell (2011) advances a well-argued defense of contextualism for predicates of personal taste and aesthetics that makes some progress with respect to the position held by López de Sa. Sundell argues, on the one hand, that impressions of disagreement or conflict as the ones we have with (1) also exist in the cases where it is clear that the asserted sentences not only do not contradict each other, but are in fact both true. On the other hand, by appealing to pragmatic and metalinguistic processes, he shows how many of the disputes of this kind can be analyzed as disputes over the selection or appropriateness of a contextually salient standard. I am sympathetic to Sundell's proposal, as I am to López de Sa's. But once more, as it stands, it is incomplete.

As indicated, a contextualist about the meaning of predicates of personal taste (and other predicates) should acknowledge that the perception of disagreement that is left in cases like (1) cannot be accounted for as a straightforward case of doxastic disagreement. For present purposes, let us accept that when two people doxastically disagree, the following inter-subjective doxastic attitude incompatibility holds:

#### 2.2.1. Doxastic attitude incompatibility

If subject *A*'s attitude is correct, then subject *B*'s attitude cannot be correct^12^.

The occurrence of doxastic disagreement justifies the disapproval of other people's doxastic attitudes. But the notion of doxastic disagreement does not play any role in a contextualist account of the remaining impression of disagreement between enlightened subjects in (1). Both utterances, Clarissa's and Jennifer's, express true propositions. Now, as López de Sa suggests, the impression of doxastic disagreement may be explained by errors about contextual presuppositions. But once we acknowledge that those presuppositions fail, the impression of disagreement should also vanish.

Thus, if in the following dialogue, Clarissa takes a visible male to be the salient one referred to by “he” in that context and Jennifer objects because she takes the salient male to be the person the previous discourse was about, any impression of doxastic disagreement vanishes when they become aware that they have different referential presuppositions.

5. (a) Clarissa: He is Scottish.
    (b) Jennifer: He is not Scottish.
    (c) Clarissa: *He* is Scottish, because the salient male I meant was not the one you have in mind but that one. [pointing to the visible person].

It is naturally possible to feel a disagreement about a “metalinguistic” proposition (concerning who is the salient male in the context, the referent of “he”), especially if participants have common knowledge about the nationalities of the visible male and the one previously spoken about, and Jennifer places a proper emphasis on her token of “he.” In this case, Jennifer's objection is similar to the one metalinguistically expressed by (6c).

Sundell (2011) resists the disagreement-based arguments of relativists that target contextualism. According to him, both intuitive impressions of disagreement or conflict, and disagreement indicated by uses of denial, or metalinguistic negation,^13^ are compatible with the absence of *some* forms of doxastic disagreement. He argues that many intuitive impressions of disagreement can be explained as cases of conflicting non-doxastic attitudes (p. 271), for instance, those manifested in this variation over (1):

6. (a) Jennifer: I really like cow's tongue.
    (b) Clarissa: Well, *I* don't like it!
    (c) Clarissa: # Nope/Nuh uh, I don't like it.

There is a perception of disagreement or conflict in (7a) and (7b), even though it is clear that the contents asserted by Clarissa and Jennifer are consistent—both are actually true. But Clarissa's disagreement with Jennifer would not have been felicitous if expressed *via* the denial in (7c). Disputes like the one in (7a) and (7b) should rather be explained by appealing to conflicting non-doxastic attitudes.

As an improvement over the notions of substantial disagreement that he discusses, Sundell proposes that we accept as (a kind of) disagreement “the relation between speakers that licenses linguistic denial” (Sundell, 2011, p. 274). Sundell gives us some examples that illustrate the variety of denial-licensing disputes. They cover presupposition disagreement [illustrated by (6a)–(6c) above], implicature, manner, character (after Kaplan, 1989), and finally context disagreement.

Context disagreement can include cases where sentences like those in (3) are uttered (Sundell, 2011, pp. 278–279). Consider this variation of the example. Adapting the point made by Barker (2002), we can imagine a case where Naomi is visiting Athens, and is curious to know what nowadays counts as rich in Greece. In reply, Didi utters (3a), “Mary is rich.” In so doing, Didi is giving “some guidance concerning the relevant standard” for richness in Greece. Barker considers these as metalinguistic uses of gradable adjectives, uses that “produce a context-sharpening effect” (Barker, 2002, p. 1) (see also García-Carpintero, 2008 for a similar discussion). If these uses exist, then we can conceive of a dispute between Didi and Naomi that concerns what the relevant standard of richness is in their context, a dispute which can be expressed by (3a) and (3b). In other words, “if context sharpening is a commonly available mode of conveying information, then a natural prediction is that such information is a possible focus of dispute” (Sundell, 2011, p. 279).

There is however a further possible kind of context disagreement. Not only can people dispute *which* is the contextually salient standard in a conversation, speakers can also dispute *which standard should be adopted*, when none is settled. There are two issues that need further explaining. One concerns the contextual disagreements where speakers dispute which contextual standard should be selected. How are such disputes to be adjudicated? A second related issue concerns rather *what drives* such disputes?

Presumably, there is nothing *prior* to some aesthetic disputes or disputes over matters of taste about which standard to adopt when *nothing* in the context settles a standard. There are no doubt culture-wide paradigms of beauty that are part of the background of many aesthetic disputes, and likewise for discussions over matters of taste, etc. But culture-wide paradigms do not suffice to resolve all such disputes. They cannot settle, for example, a disagreement over who is more truthful to nature, Turner or the pre-Raphaelites.

Where nothing prior settles a dispute, a plausible hypothesis to explain the *persistency* of a disagreement is that in those cases conflicts of pro-attitudes merge with contextual disagreements (discarding other explanations for persistence, such as lack of knowledge of the nature of the dispute, of the relevant background, individual stubbornness, etc.) In a dispute of the kind now contemplated, each speaker tries to impose her own standard as the salient standard of the context, insofar as the speakers are motivated to push their own standard. But *why* should anyone do so? In other words, why would anyone want her own perspective on the things she appreciates (or doesn't) to be the perspective that others also have about what they appreciate (or don't)? If we assume that there is reason to treat different perceptions of taste as equally veridical, it becomes evident that an explanation of how these different perceptions can ground conflicts is missing from most of the recent literature on these issues.

### 2.3. Conflict

Huvenes (2012) discusses examples similar to (7), “I like cow's tongue”/ “Well, I don't!.” He considers whether examples of this kind [and others, like (1)] admit linguistic denials, and other markers of disagreement like “that's not true”/ “that's false” or “I disagree.” He argues that considerations having to do with disagreement do not undermine contextualism. Like Sundell, Huvenes also considers that there are a variety of forms of disagreement. He tries to defend the idea that two people can disagree, even if they *both speak truthfully*. These are the cases like (7), where speakers voice their different dispositions toward given foods. Huvenes mentions that the idea of appealing to conflicting pro-attitudes, desires or preferences, is not original. His idea is to use the distinction (Stevenson, 1963) made between “disagreement in belief” and “disagreement in attitudes,” i.e., between doxastic and non-doxastic disagreement. Although the idea of conflicting conative attitudes is assumed to play a role in conflicts over evaluative matters in general, it is seldom explained.

The first chapter of Stevenson (1963) is dedicated to the nature of ethical disagreement, and the book starts by drawing the above mentioned distinction between doxastic and conative attitude disagreement, a distinction that philosophers, but mostly meta-ethicists, have assumed to exist ever since it was made. Expressivists (Stevenson, 1963, Blackburn, 1984 or Gibbard, 1990), relativists (Egan, 2012; MacFarlane, 2014), and contextualists (Sundell, 2011; Huvenes, 2012; Marques and García-Carpintero, 2014; Marques, Unpublished Manuscript, etc.) all embrace it.

We are concerned with the possibility of conflicting conative attitudes accounting for the resilient impressions of disagreement that most theorists argue exist in the cases under consideration. How should conflicting attitudes be explained? Two hypotheses for the conditions under which attitudinal conflicts occur have been put forward in the literature. The first condition is one of subjective rationality, and the second is one of satisfaction.

The rationality condition is what Kölbel as in mind when he describes disagreements thus: “we could not rationally accept what the other has asserted without changing our minds” (Kölbel, 2004, p. 305). The nature of the modality would need elucidation. Moreover, attitudes that are not beliefs, i.e., are non-doxastic, seem to raise further difficulties for a rationality constraint.

The satisfaction condition is what Stevenson has in mind with that sense of disagreement that “involves an opposition of attitudes both of which cannot be satisfied” (Stevenson, 1963, pp. 1–2). The two conditions can be summarized as follows.

**Rationality:** It is not possible for an individual to rationally have a pair of attitudes *X* and *Y* just in case there is an attitudinal conflict between subjects *A* and *B* when *A* has attitude *X* and *B* has attitude *Y*.

**Satisfaction:** If a subject *A*'s attitude can be satisfied, then *B*'s attitude cannot be satisfied.

We may however have reasons to doubt that RATIONALITY is true. It is not clear whether it is ever irrational to have a pair of conative attitudes like desires, or certain emotions (love and hate, fear and hope, say). In his *Treatise*, Hume argued that

it is only in two senses, that any affection can be called unreasonable. First, When a passion, such as hope or fear, grief or joy, despair or security, is founded on the supposition or the existence of objects, which really do not exist. Secondly, When in exerting any passion in action, we chuse means insufficient for the designed end, and deceive ourselves in our judgment of causes and effects. Where a passion is neither founded on false suppositions, nor chuses means insufficient for the end, the understanding can neither justify nor condemn it

Hume (1978, pp. II, iii, 3, 415).

Both senses support the idea that the “unreasonableness” of the passions depends on the possibility of their satisfaction (whether their objects exist, and whether the means to attain them are sufficient). If Hume is right, then the individual rationality constraint for conative attitudes depends on an individual satisfaction condition. It is hence conceivable that someone is “not unreasonable” for having two attitudes *X* and *Y*, even if there is an attitudinal conflict between *A*'s attitude *X* and *B*'s attitude *Y*. Since we are left with SATISFACTION as the real condition on the rationality of attitudes, a question arises as to how it impacts on the existence of inter-personal conflict.

For SATISFACTION to be an acceptable condition for conflict, more has to be said about *why* certain pairs of attitudes, when held by two or more people, give rise to conflicts. Simply mentioning that two attitudes cannot be both satisfied will not account for many of the conflicts arising from the manifestation of different dispositions. In other words, there are pairs of attitudes held by different people that can be satisfied and *nonetheless* the people at stake seem to be in conflict. If the conative attitudes expressed are like those conveyed in (7) “I like cow's tongue,” and these are strictly individual dispositions, then clearly the attitudes conveyed can be both satisfied. Since both dispositions or desires toward cow's tongue can be satisfied—Jennifer can eat what she desires and Clarissa can refrain from eating what she doesn't desire—there seem to be no grounds for those attitudes to be in conflict or incompatible, apart from the fact that they are *different*^14^.

On the other hand, having different desires, or desiring different things, can't be a basis by itself for conflict or disagreement, as this example clearly illustrates: Jennifer quite fancies Ferrán Adriá, but Clarissa fancies his brother Albert instead. There's no conflict there, surely. Difference in attitudes does not establish conflict.

In what sense are Jennifer's and Clarissa's different dispositions toward cow's tongue in conflict? As long as they can concur in not forcing their choices on each other, both can have their preferences satisfied. Yet presumably we *may* still hear a disagreement in straightforward expressions of preferences like (7). An appeal to different individual dispositions by itself does not explain *why* even in this case we hear them disagreeing. If each of them is expressing a personal preference, with no consequences for what the other will eat, where is the remaining conflict?

Given that we have dismissed RATIONALITY, and that SATISFACTION seems unsatisfactory if the attitudes at stake are purely *first-personal singular*, it seems to follow that we can only read (7) as expressing conflict between two people insofar as we see it as an expression of an expected common disposition shared by Clarissa and Jennifer. And unless we have a good explanation of *why* having the same dispositions matters, we will be incapable of explaining why people with different desires, preferences, or dispositions, have incompatible attitudes, or of explaining the role of conative attitudes in conflicts about evaluative thought and discourse, for instance in cases like (1)^15^.

A theorist that aims to account for evaluative dispositional properties should answer several questions. (I) Are the dispositional properties first-order or higher-order? (II) Are the dispositions first-personal singular or plural? And (III) what is the nature of the dispositions at stake?

I am inclined to opt for the higher order nature of these dispositions, because of examples of the following sort: Suppose I have a terrible cold. I've lost my sense of smell and taste. I'm offered a dish that has been prepared by the chef at my favorite restaurant. There's nothing he cooks that I don't like, so although I have not tried this one dish, I am almost certain it is delicious. But the dish does not taste like anything to me now (and I have never tried it before). It is not incoherent to believe “this does not taste delicious to me now, but I know it is delicious.” *Mutatis mutandis* for something cooked by a hypothetical friend with terrible taste and poor hygiene habits. It is also, in similar conditions, not incoherent to believe “this does not taste bad to me now, but it is disgusting.” This speaks at least in favor denoting by “disgusting”/ “delicious” whatever my gustatory experience would be in ideal conditions, or at least in normal conditions.

Are these dispositions first personal singular or plural? How can I generalize from the cook at one of my favorite restaurants and the hypothetical friend with bad taste? Presumably my generalization encompasses not just why the restaurant is good for me (in normal or ideal conditions), or why my friend has terrible taste (for me in normal or ideal conditions) but for anyone who is *sufficiently like me in relevant respects* (in constitution or in cultural background, or whatever turns out to be the relevant respects). But there is a further possible variation here, depending on which evaluative property is expressed: who “we” designates may vary from a large group—possibly everybody, to a very small group—oneself only. Finally, what is the nature of the dispositions at stake? The attitudes at stake may be desires, but presumably they could be other more primitive emotional reactions.

The hypothesis that “disgusting” expresses higher-order plural dispositions, and not just first-personal singular first-order dispositional responses seems to be confirmed by Rozin and Fallon's work:

The notion that disgusting items taste bad may be problematic. Whereas most people have never tasted most things they find disgusting, they are convinced that these substances would taste bad. Of course, bad refers not to sensory properties but to their interpretation of them. Thus, even if ground dried cockroach tasted just like sugar, if one knew it was cockroach, this particular sweet powder would taste bad… It is the subject's conception of the object, rather than the sensory properties of the object, that primarily determines the hedonic value. Although certain strong negative tastes (e.g., bitter tastes) may not be reversible by manipulation of the object source or context, we suspect that any positive taste can be reversed by contextual or object information (Rozin and Fallon, 1987, p. 24).

Now, David (Lewis, 1989) offers a schematic definition of what a value is:

[S]omething of the appropriate category is a value if and only if we would be disposed, under ideal conditions, to value it (Lewis, 1989, p. 68).

To value something is, for Lewis, to be in a certain sort of motivational mental state: to desire to desire it. This guarantees the internalist connection between value and motivation. Values are the things that *we* are disposed to desire to desire in certain circumstances. There are two categories of such things: the states of the world we desire to be the case, i.e., the propositions we desire to be true. These are *de dicto* desires. And we also desire to *be* in a certain way. These are *de se* desires. Lewis's dispositionalist theory fits well with the kind of relational account of evaluative properties described by analogy with the color case in §2. On this theory, to find that cow's tongue is tasty is to be disposed in the right way toward cow's tongue, i.e., it is to value *having pleasant gustatory experiences when eating cow's tongue*. And to find that cow's tongue is disgusting is to be disposed in the right way against cow's tongue, i.e., to value *not being in contact with cow's tongue*.

On the Lewisian theory, the evaluative property expressed involves the relevant group to which the speaker belongs. It is, if we want, a first-person plural secondary property, or a *de nobis* secondary property. The theory offers further advantages. It is cognitivist, since it accounts for the evaluative property expressed by the value predicate or word—and it can be true or false that cow's tongue is tasty (or disgusting), and even that Jennifer (or Clarissa) can be mistaken about cow's tongue being tasty or not. At the same time, the theory is sufficiently subjectivist and dependent on people's desires to accommodate the perceived importance of conative attitudes in disputes of taste^16^.

Group membership for the purposes of identifying the relevant evaluative properties expressed by value terms cannot be the sort of thing that depends exclusively on one's occurrent desires. One may be mistaken at a given moment about one's overall dispositions, and one's occurrent desires may be affected by extraneous causes. If this occurs in the personal case, *a fortiori* it can happen in the first-person plural case, and one may be mistaken at any given time about what one's group values. Group identity and membership cannot depend exclusively on one's conversational interlocutors at a given moment. Because of this, what *we* (i.e., me and people sufficiently like me in the relevant respects) find delicious or disgusting is not determined by intra-conversational contextual factors, or at least, not entirely.

If the disagreement in (1), as in (7), results from conflicting dispositions and is about which standard should be adopted, what exactly drives Clarissa and Jennifer to try to impose their own standard? The previous paragraph indicates various ways the “selection of a standard” or a “dispute over a standard” can take place: people may be mistaken about what standards they actually endorse, they may be mistaken about group membership (who are “we”) or it may simply be indeterminate who “we” are, or how “we” are to respond in ideal conditions. If and when subjects are disputing which standard *should* be adopted, they are disputing what *they* collectively should be disposed to (dis)value.

To repeat, on a dispositional account of value along the lines of Lewis's, a standard of taste is a kind of dispositional property, the disposition to value certain things. The dispositions at stake are first-person plural. This should yield the desired result. Clarissa finds cow's tongue disgusting. The theory should ascribe to her the disposition to value, i.e., to desire *that we desire not to eat cow's tongue*. Jennifer, however, desires *that we desire to eat cow's tongue*. Clarissa and Jennifer's desires amount to a disagreement in attitudes because they cannot be jointly satisfied at the same world.

A remaining question is the following: Why does it matter that people share a common value standard? In particular, why does a shared standard matter for *tasty* or *disgusting*, but not for *fancying*?^17^ The next section tries to offer an answer to this question, relying on the role of coordination on the evolution of the relevant dispositions.

## 3. Coordination

The beginning of a solution should take coordination into account. Coordination plays a role in a different sense of *agreement* to the ones discussed so far—namely, in the sense of an agreement as a convention. For (Lewis, 1969), conventions are solutions to coordination problems. Lewis follows Hume's account of convention and agreement in the *Treatise*:

It is only a general sense of common interest… I observe, that it will be to my interest [e.g.,] to leave another in the possession of his goods, provided he will act in the same manner with regard to me. When this common sense of interest is mutually expressed and is known to both, it produces a suitable resolution and behavior

(Hume, 1978, pp. III.ii.2,490).

What connects coordination with the kind of *de nobis* dispositions claimed to be central in evaluative properties?

Bacharach (2006) and Gold and Sudgen (2007) have done a considerable amount of work on the role of first-personal plural intentions in decision-theoretic reasoning irreducibly involving groups with which agents identify^18^. These dispositions are essential for group cohesion. Let us call them “*de nobis* dispositions.” When Clarissa and Jennifer have *de nobis* dispositions, there is an increased probability that their actions will be coordinated with respect to an indefinite plurality of projects. An explicit indication that the presupposition of commonality (see López de Sa, 2008) fails, as in a metalinguistic expression of disagreement over the relevant standard (see Sundell, 2011), manifests the absence of such common *de nobis* dispositions, and it may undermine group cohesion. *This* is the practical aspect that is missing in other semantically similar cases, such as the disagreement about being rich, or who “he” refers to. And it explains where the conflict of attitudes arises.

I next offer a conjecture as to why we have such *de nobis* dispositions.

### 3.1. A conjecture

The conjecture advanced here involves various components. The first is a commonly shared assumption among evolutionary cognitive scientists, namely that various kinds of coordination problems are at the root of our having, as humans, evolved to have the dispositions we have. The second component connects this evolutionary assumption with dispositional theories of value, such as Lewis's. As a result, value judgments about matters of taste, aesthetics or morality are such that they both express dispositional properties and, crucially, reveal conflicts when dispositions vary. The present conjecture seems to be confirmed by research in biology, evolutionary psychology and anthropology. The conjecture, to be clear, is that our preference for *some* converging dispositions, and our aversion to *some* diverging dispositions, has an evolutionary explanation connected with finding needed solutions to recurrent coordination problems.

The conjecture is corroborated for instance by Tooby and Cosmides's work. Our distinctive capacity for cooperative behavior was, they have argued, evolutionarily important for human survival. Tooby and Cosmides (2010) summarizes many of their results. According to them, alliances pose a “series of adaptive problems that selected for cognitive and motivational specializations for their solution” (p. 200), where the two biggest obstacles to alliances are the problem of free-riders and the problem of coordination. Coordination to achieve common goals is necessary for coalitions, and it is also necessary that cooperators are not outcompeted by free-riders. We have evolved both anti-free rider adaptations and coordination adaptations. Tooby and Cosmides indicate that adaptations for coordination include programs implementing

a theory of group mind; programs implementing a theory of interests; programs implementing a theory of human nature; programs for leadership and followership; the outrage system; theory of mind; co-registration programs for solving common knowledge problems; language; and an underlying species-typical system of situation representation which frames issues in similar ways for different individuals

(Tooby and Cosmides, 2010, p. 202).

Sharing the same evolved architecture, they claim, provides a partial foundation for resolving the game theoretic problem of common knowledge with finite cognitive resources. For cooperative action to be taken, evolved procedures must exist for inducing or recognizing sufficient coordination in situation representation.

Among the adaptations that contribute to coordination are our emotional responses. Specific emotions are evolved systems of internal coordination, activated in response to evolutionarily recurrent situations such as danger, contamination, conflict or pleasure.

More generally, there seems to be a psychophysics of mutual coordination and coregistration, involving (for example) joint attention and mutual gaze, especially timed when salient new information could be expected to activate emotional or evaluative responses in one's companions. The benefits of coregistration and mental coordination can explain (at least in part) an appetite for co-experiencing (watching events is more pleasurable with friends and allies), the motivation to share news with others, for emotional contagion, for gravitation in groups toward common evaluations, for aversion to dissonance in groups, for conformity, for mutual arousal to action as with mobs (payoffs shift when others are coordinated with you), and so on.

(Tooby and Cosmides, 2010, p. 205).

The research about the evolution of taste and disgust, the education of taste, and eating customs, illustrates this broad description of the importance of coordination in human cognition. I mention briefly the case of what is disgusting, after (Rozin and Fallon, 1987; Rozin, 1996). Here is a very short summary of the explanation. As omnivores, humans have a very varied diet, but this means that they are at a high risk of consuming toxic substances. The evolution of gustatory taste permits discriminating potentially edible things. According to Rozin, disgust is the fear of incorporating an offending substance into one's body. The things that humans find disgusting things are, mostly, those coming from animals (in particular, some animal parts, like tongues and other internal organs). But there is a problem: it seems there is a wide variability in what is found disgusting (and conversely, tasty) from culture to culture, which suggests that there is a crucial learning period. Elizabeth (Cashdan, 1994) argues that there is indeed a sensitive period for learning about food in the first 2–3 years of a child's life. After 3 years, children's tastes diminish drastically. Coordinating eating habits with those of the immediate group may be one of the first requirements for survival. It then becomes a way of identifying one's group and community.

Pinker (1997) discusses the significant case of food taboos. According to him, food taboos indicate that the coordination of eating habits with those of one's group is important because it contributes to strengthening the cohesion of the group. Being able to eat together may permit the formation of new alliances. The feast days of many religions have as a central component rituals involving food and “breaking bread together” (Pinker, 1997, p. 385).

Now, conflicts may occur in actual situations where coordination toward common goals may be hindered—for instance, when Clarissa and Jennifer cannot agree on what *they* should eat. On the other hand, conflicts may occur in evolutionarily recurrent situations that have posed coordination problems, and thus led to the selection of specific emotional responses (responses toward edible things, toward dangerous or pleasurable situations, toward other people or their actions).

The conjecture here advanced is that in cases of this kind—where sharing the relevant dispositions has played a role in finding coordination solutions in recurrent situations—the existence of divergent emotional responses is perceived as signaling potential conflicts, and thus ground the conflict among conative non-doxastic attitudes: *not all* of their desires will be satisfied.

The conjecture is illustrated by the case of tasty and disgusting things. Being disposed to eat the same sort of things enables further cooperation and altruistic behavior, and is more likely to lead to future benefits. Humans have evolved to approve of others with similar dispositions, and have evolved to disapprove of others with dissonant dispositions. Not being similarly disposed in some relevant aspects may hinder further cooperation. The desires that concern the benefits that result from others' cooperative behavior toward oneself may fail to be satisfied.

This research supports the claim that humans have a preference for consonance and an aversion to dissonance in *certain* kinds of dispositions. Other research supports the claim that certain modes of cognition are first-person plural or *de nobis*. Frith and Frith (2012) have recently reviewed the recent work in cognitive science and psychology on the various “mechanisms of social cognition.” Among such mechanisms are, for instance, empathy or emotional contagion that permit alignment of representations, as well as forward modeling that allows the prediction of other's behavior. Some of the neural mechanisms involved in the observation of others and in learning, at the implicit level, are association, reward, gaze following and mirroring. Ongoing research on these mechanisms of social cognition is revealing the role they play in learning, cooperation, and language acquisition.

## 4. Consequences

How does the conjecture fir in with (i) the dispositional account of values *a la* Lewis that is being here assumed, and with (ii) a contextualist semantic account of evaluative predicates in general?

The Lewisian theory is not only internalist and cognitivist, but it is also naturalist. Values are dispositional states. If any dispositional theory is correct, it has to fit with what the best theories of the natural and social sciences tell us about the relevant kind of dispositions. Evolutionary psychologists' work on the evolution of altruism and cooperation, and on the evolution of the sense of taste for instance, corroborates a dispositional theory, at least with respect to taste properties. It may be that further research on the mechanisms of social cognition will tell us more about the *x* character of such dispositions.

This paper started with a discussion of challenges to contextualist semantics. The issue was whether a contextualist semantic account of evaluative predicates like “tasty,” “disgusting” (and others more robust than taste predicates) can accommodate and explain disagreement data. A contextualist semantics that respects the metaphysical view of evaluative properties as secondary or dispositional properties of the sort discussed here will allow for the possibility that two speakers may be in dispute over different evaluative properties. One speaker may express a property about group_1_ to which she belongs, i.e., that it values *X*, and another speaker expresses a property about group_2_ to which she belongs, i.e., that it does not value *X*. Both speakers may be speaking truly. Wasn't this the main objection to contextualist accounts, that there seems to be some sense of disagreement left that cannot now be captured by the semantics? On a dispositional theory like Lewis's account, we have an explanation that covers doxastic disagreements, as well as an explanation of conflicting desires, where such a conflict exists if and when interlocutors are members of the same group. This could however mean that the challenge of lost doxastic disagreement results in a challenge of lost conflict of attitudes too. Or does it?

Contextualists have appealed to presuppositions of commonality to deal with the challenge of lost disagreement—we suppose that our interlocutors are like us in the relevant respects. They have appealed to metalinguistic disputes about the selection of standards—even if we are both speaking truly, we may in fact be engaged in a dispute over what standard should be implemented. And they have moreover appealed to conflicting conative attitudes that in any case remain. The main aim of this paper was to show the need to say more about these kinds of explanation. What drives disputes over the selection of evaluative standards, and why does it matter that common standards be accepted? What makes it the case that a pair of conative attitudes are in conflict? What is the role of coordination in finding common standards and in attitudinal conflicts? Keeping in line with the naturalistic motivation for a dispositional account of value properties, the paper has offered a brief review of some of the central research in evolutionary psychology and cognitive science that can begin to fill in the blanks, connecting on the one hand dispositions that help to find solutions to recurrent coordination problems, and on the other hand evaluative thought and discourse in general. Does this put us in a better position to answer the challenges of disagreement and conflict?

On the contextualist account, Clarissa and Jennifer express distinct but equally true propositions. This offers a semantic implementation of the relational account of the dispositional properties expressed by taste predicates. But contextualists about predicates of taste and value face the challenge of explaining how two people can accept different true propositions and nonetheless disagree. The suggestion here offered tried to develop the proposals put forward by López de Sa, Sundell, Huvenes, and Marques and García-Carpintero, by offering what the appeals to presuppositions of commonality, metalinguistic disputes and conflicting non-doxastic attitudes were missing.

The impression that there is a doxastic disagreement could presumably be explained by the existence of folk invariantist semantic intuitions. But there seem to be resilient disagreements even where semantically informed speakers like Clarissa and Jennifer still insist on uttering sentences like (1a) “Cow's tongue is disgusting” and (1b) “No, it isn't, it's delicious!” These can be presumably explained as metalinguistic disagreements over the selection of an appropriate standard, as Sundell proposes. What distinguishes cases like (1) from cases where speakers simply express their individual preferences, like (7), is that the former cases trigger presuppositions of commonality that the latter do not. But other cases of presupposition failure do not generate disagreements; in fact, learning that a presupposition fails usually *dispels* disagreements. We can anyway assume that a conflict of attitudes remains. If these attitudes were simply the expression of individual desires, and since two people with different personal desires can both be satisfied, it is hard to see what the cause of the remaining conflict can be. I have offered a broader explanation of conflicting conative attitudes, in line with a dispositional theory. However, this explanation still leaves us with a problem: if, on assumption, what Clarissa and Jennifer say is true with respect to their respective standard (which concerns two distinct groups), and if their non-doxastic attitudes are in conflict only if they concern the same group, then we still do not have an explanation of the conflict of attitudes.

In Section 2.3, I pointed to the fact that group membership cannot be the sort of thing that depends exclusively on one's occurring desires. One may be mistaken at a given moment about one's overall dispositions, and one's occurring desires may be affected by extraneous causes. Moreover, one may be mistaken at any given time about what one's group values. Also, group identity and membership cannot depend exclusively on one's conversational interlocutors in a context. It is not that whatever is a value is whatever the interlocutors in a conversation are disposed to value; context contributes to determine *which* value property is *expressed in a context*. As a simplified illustration, context determines whether by “tasty” the interlocutors mean *tasty for people with sophisticated gourmet training*, or *tasty for the typical north-European 3 year old child*. But a conversational context does not constitute the value property itself. The property at stake, whatever it is, is whatever the relevant group is disposed to value in the right conditions.

The concern that contextualist explanations are limited to intracontextual disputes does not arise straightforwardly. Because the relevant group's identity, membership and composition are not context-dependent matters, whether or not there is a disagreement or a conflict of attitudes is not straightforwardly a result of whether two people participate in the same conversation. Rather, it is a result of whether their doxastic or conative attitudes are compatible or in conflict. Finally, group identity and group membership may be indeterminate. This indeterminacy, together with some indeterminacy concerning what *we* should do in ideal conditions of full imaginative acquaintance, leaves ample room for meaningful disputes about evaluative matters, and for metadisputes about what values we should share.

In the previous section, I reviewed some work that shows the importance of common evaluations for cooperative projects. The possibly variable extension of a given group (and the indeterminacy of the group identity and extension in question), together with “the benefits of coregistration and mental coordination” can at bottom be the reason why, even when people have different standards, they strive to establish a common ground, or, to put it another way, to extend group membership. Attitudinal conflicts can endure wherever there are expectations concerning what *we*, together, should come to value.

This is, in summary, a rephrasing of Lewis's conditionally relative view. There is no absolute answer as to who *we* are: “What I mean to commit myself to is conditionally relative: relative if need be, but absolute otherwise” (Lewis, 1989, p. 85).

### Conflict of interest statement

The author declares that the research was conducted in the absence of any commercial or financial relationships that could be construed as a potential conflict of interest.

## Footnotes

^1^See for instance (Glanzberg, 2007; Stojanovic, 2007; Rosenkranz, 2008; Schaffer, 2009; Coliva and Moruzzi, 2012; Marques, 2014a,b).

^2^See (López de Sa, 2008; Marques and García-Carpintero, 2014).

^3^See (Sundell, 2011).

^4^See (Sundell, 2011; Huvenes, 2012; Marques and García-Carpintero, 2014).

^5^*De nobis* are plural *de se* attitudes. Where *de se* attitudes are specific kinds of attitudes or mental states about oneself, *de nobis* attitudes are a specific kind of attitudes or mental states about ourselves. There are well-known motivations for *de se* thought, and different theories that try to accommodate what is essentially *de se* in thought. In Marques (Unpublished Manuscript), I draw a parallel between *de se* and *de nobis* attitudes by showing that the same reasons that support the existence of a distinctive kind of first-personal attitudes can be replicated for the first-personal plural case.

^6^See for instance (Egan, 2012). The present paper can be seen as offering a justification for maintaining the more classic dispositional theory, instead of the relativist modification offered by Egan. In Marques (Unpublished Manuscript), I argue that Egan's *de se* version of dispositionalism about values fails to accommodate conflicting attitudes, and, given the nature of the theory, it also fails to accommodate doxastic disagreement.

^7^The example honors Clarissa Dickson Wright and Jennifer Paterson, the *Two Fat Ladies*.

^8^Assuming subject-relative standards here plays a dialectical role. Many authors in the literature assume subject-relative standards, and most objections to relativism focus on individual standards of taste.

^9^Baker (2012) criticizes this proposal. He invokes three commonly accepted tests for presuppositions (cf. von Fintel, 2004), and points out that they do not appear to support López de Sa's claims. For discussion, see (Marques and García-Carpintero, 2014); the presentation of the discussion in this section summarizes our work in that paper.

^10^Kennedy (2007) and Kennedy and McNally (2010), for instance, argue for a contextualist treatment for relative gradable adjectives such as “tall” or ‘rich,” although not for absolute gradable adjectives like “spotted” or “full.” Thanks to an anonymous referee for pointing this out.

^11^It might be questioned that the impression of disagreement is anyway resilient in the dialogue between Clarissa and Jennifer even for semantically enlightened subjects, after Jennifer says, for instance, “Cow's tongue is delicious given what counts as delicious for me; I see that you have different tastes.” Perhaps it is not obvious that there is a resilient sense of disagreement, but I am taking as veridical the reports given by many people that even after a qualification of this kind is made, they still perceive a conflict between Jennifer and Clarissa.

^12^For more on doxastic disagreement and exclusion, see (Marques, 2014a).

^13^See (Horn, 1989; Carston, 1998, 1999).

^14^Schroeder (2008) criticizes several versions of expressivism for failing to explain, and merely assuming, that pairs of different conative attitudes are incompatible, or inconsistent. He says “I think that none of these looks remotely satisfactory as an expressivist explanation of why ‘murdering is wrong’ and ‘murdering is not wrong’ are inconsistent. None answers the basic question of what makes disapproval and tolerance of murdering inconsistent with one another. Each posits *that* there are such mental states that are inconsistent with one another, but none explains *why*” (Schroeder, 2008, p. 587). I agree with Schroeder's criticism of expressivism. Contextualists and relativists should be careful not to make the same mistake.

^15^For discussion and examples illustrating the need for a good theory of conflicting conative attitudes, see (Lewis, 1989) and (Marques, Unpublished Manuscript).

^16^Anonymous referees pointed out that there seems to be a difference between clearly evaluative predicates (moral terms for instance) and many taste predicates of the kind discussed here. Although we may expect convergence, they suggested, it would not be plausible to claim that “delicious” or “disgusting” express *de nobis* dispositional properties. I admit that there may be some cases where apparent taste predicates express *first-personal singular*, i.e., *de se* properties: a disposition to have a certain response or reaction in the presence of certain substances. The present account can be seen as defending an outline of the conditions a theory must satisfy if it is to be *evaluative* and to allow for *conflict and disagreement*. Singular *de se* dispositional properties can still be evaluative, but not allow for conflict or disagreement. Singular first-order *de se* dispositional properties will not be evaluative nor allow for conflict or disagreement.

^17^As an anonymous referee pointed out, “tasty” and “disgusting” are adjectives whereas “fancies” is a verb. I don't think this affects the main point. The background story for taste adjectives of this kind is that they express certain kinds of dispositions, namely dispositions that envolve desires. “Fancies” expresses the occurrence of a given desire. The main point here is that the desires at stake appear to be satisfiable and hence there's an explanation of the conflict missing. The example with “fancies” could be changed to an example with an adjective, for instance “simply irresistible.”

^18^See also (Marques and García-Carpintero, 2014).
